# Chyloptysis with chylopericardium, a rare case and mini-review

**DOI:** 10.1186/s12890-018-0583-y

**Published:** 2018-01-29

**Authors:** Xuming Luo, Zhuhua Zhang, ShiQiang Wang, XianDong Gu, Xiongbiao Wang

**Affiliations:** Department of Respiratory Medicine, PutuoHospital, Shanghai University of Traditional Chinese Medicine, Shanghai, No.164, LanXi Road, Shanghai, 200062 China

**Keywords:** Chyloptysis, Chylous disorder, Lymphangiography

## Abstract

**Background:**

Chyloptysis is reflux of chyle into the lungs and is a rare manifestation of primary chylous disorders.

**Case presentation:**

Over 29 months, on the basis of x-rays, a 33-year-old female was repeatedly misdiagnosed with recurrent right-sided pneumonia; her symptoms included a recurrent cough, the appearance of cheese-like sputum, and diffuse pulmonary exudates. There was a history that abundant fluid in the pericardium was confirmed with echocardiography to have been present and asymptomatic for 4 years. Lymphangiography and surgery confirmed that the terminal portion of the thoracic duct was compressed by thick fibrous tissue and the vascular sheath of the internal jugular vein. Chyloptysis caused by high peribronchial lymphatic pressure was diagnosed and surgical intervention relieved the symptoms.

**Conclusion:**

Chyloptysis is rare and easy to misdiagnose but is a typical symptom of chylous reflux syndrome.

## Background

Chylous disorders are uncommon; chylous ascites, chylothorax, and chylopericardium are relatively more frequent, and chyloptysis is extremely rare. Chyloptysis is defined as the expectoration of milky-white sputum rich in chyle. Initially 11 cases reported in the literature were reviewed in 2012 by Kato [1]; no more than 20 cases have been reported overall [2–8]. Patients and primary care physicians often do not notice this sputum type, which can lead to misdiagnosis [9]. Here we described one such case that persisted for 29 months until we confirmed and corrected the disorder.

## Case presentation

**Fig. 1:**
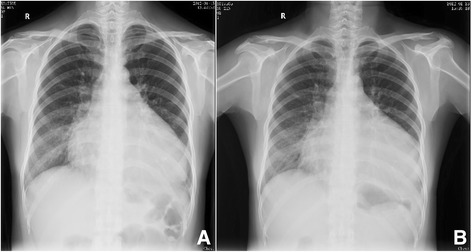
Chest x-ray of pneumonia (**a**) obtained on January 15, 2012, and (**b**) x-ray from January 23, 2012

**Fig. 2:**
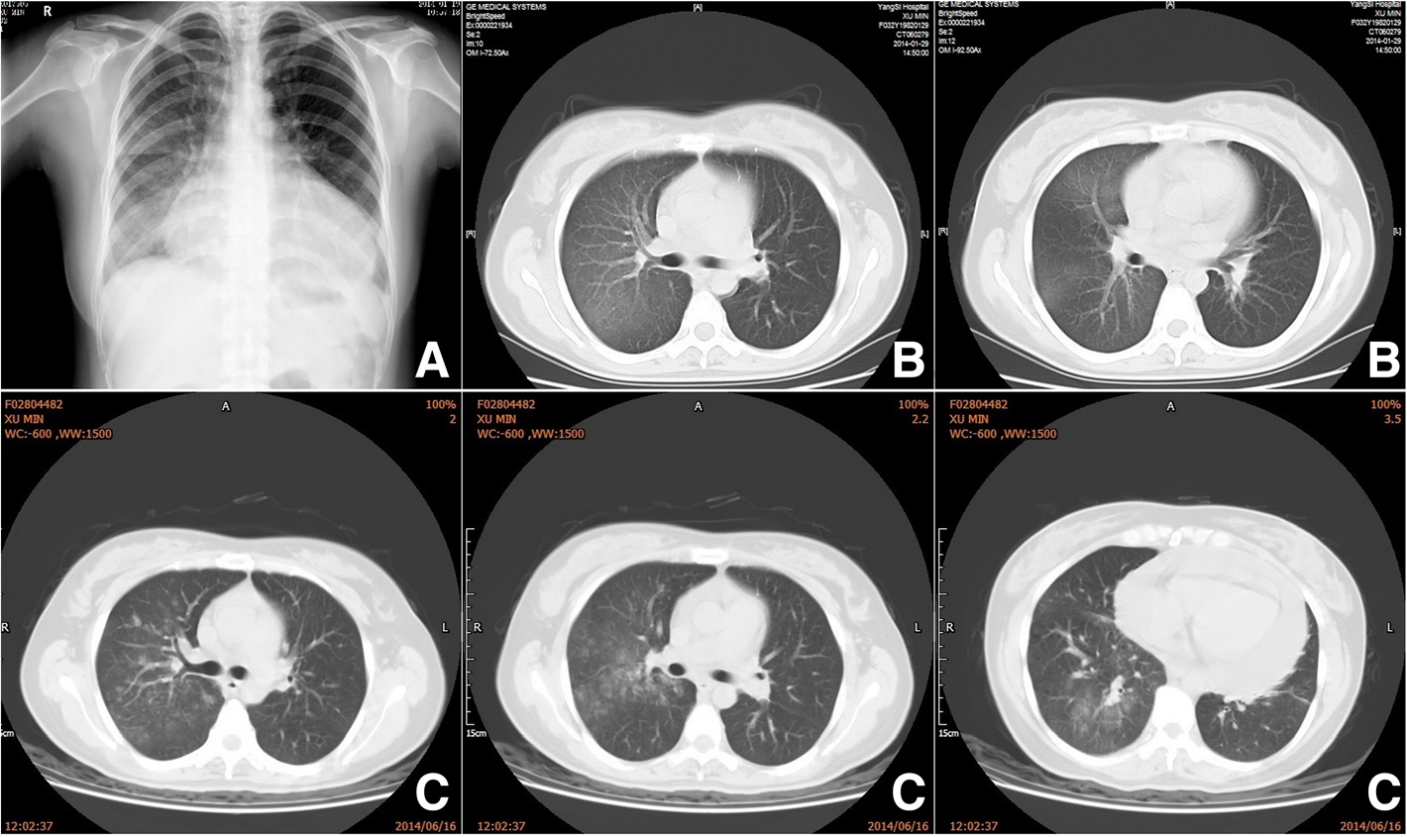
Chest x-ray and CT image of the chest obtained at admission showing ground-glass opacities in the right lobes. **a** Chest x-ray obtained on January 19, 2014; (**b**) CT images of the chest obtained on January 29, 2014; (**c**) CT image obtained on June 16, 2014

We describe a 33-year-old female with an enlarged heart and copious fluid in the cavum pericardii, as confirmed by echocardiography, although she had been asymptomatic for 4 years. In January 2012, she presented to the hospital with a severe cough and cheese-like-sputum mixed with blood but no fever. X-ray examination suggested that there was a diffuse exudate in the right lung but no tumor mass (Fig. 1a). Leukocytes and C-reactive protein (CRP) were within the normal range. Pneumonia was diagnosed and antibiotics prescribed. Symptoms eased after 1 week but there was no obvious change in the chest x-ray (Fig. 1b). The patient presented to the hospital again in November 2013 and 6 more times between November 2013 and June 2014. The “pneumonia” in the right pulmonary lobes persisted. According to the chest x-ray, the exudates decreased but did not disappear between episodes (Fig. 1 and Fig. 2a, b). Earlier, high-resolution computed tomography (HRCT) did not suggest lymphangioleiomyomatosis and showed no bronchiectasis. Upon examination, the patient did not have yellow dystrophic nails, as are often seen in yellow-nail syndrome. She had no other previous medical, social, or family history to suggest a diagnosis.

**Fig. 3:**
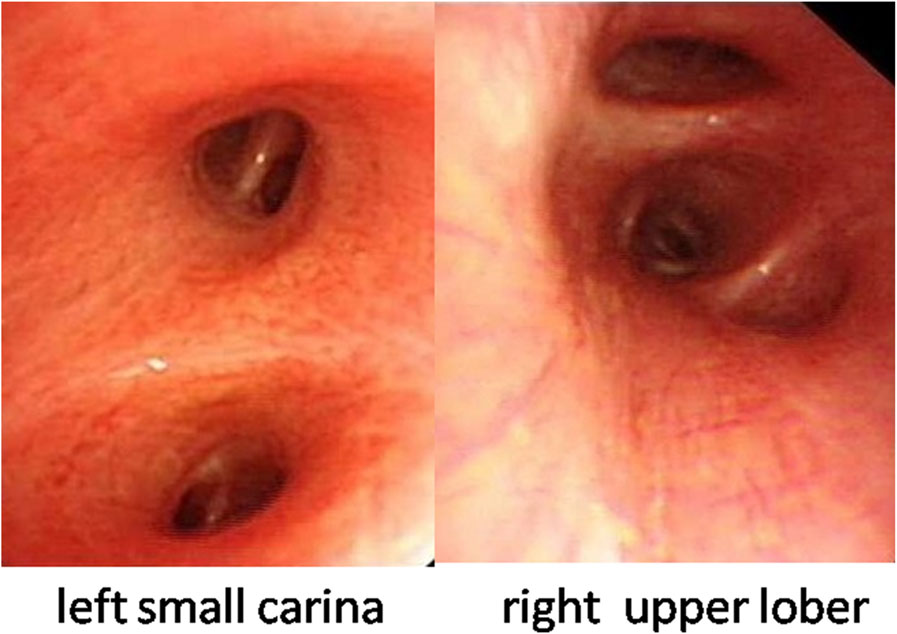
Bronchoscope found hyperemic friable mucosa

**Fig. 4:**
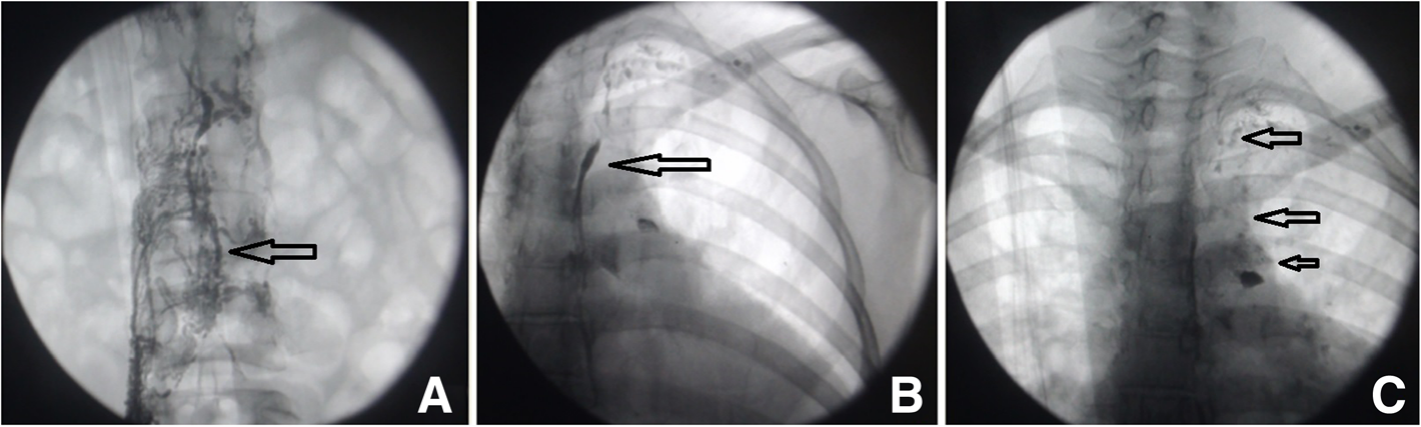
Lymphangiography showing (**a**) dilated and tortuous right iliac and retroperitoneal lymphatic vessels, indicating a structural disorder, and a cistern in the second lumbar area; (**b**) dilated ectatic proximal thoracic lymphatics; and (**c**) visible reflux of contrast medium into the left neck trunk, left subclavian trunk, and left bronchomediastinal lymphatictrunk

During a hospital visit in June 2014, the patient’s CT scan still presented diffuse exudation in the right lung (Fig. 2c). Her erythrocyte sedimentation rate, CRP, and other labs (antinuclear antibody, double-stranded DNA [dsDNA], histones, U1-snRNP/Sm, SS-A 60 kD, Ro52, SS-B, Scl-70, cardiolipin/ACA, Jo-1, ribosome/PO, nucleosomes, proliferating cell nuclear antigen, and Pm-Scl) were negative. Mucous hyperemia of the bilateral bronchi and a milky-white sputum in the lumen was confirmed with bronchoscopic examination (Fig. 3). Bronchoalveolar lavage fluid (BAL) was bloody and positive for Sudan III stain. Triglycerides were high (7.91 mmol/L). A biopsy of the right lung via bronchoscope showed normal tissue with some cellulose exudates and squamous epithelium. Apirated pericardial fluid was orange and chylous; a chyle test using Sudan III stain was positive. Laboratory data included leukocytes (1.21 × 10^− 9^/L, mononuclear cells 90%), triglycerides (18.72 mmol/L) and total cholesterol (2.07 mmol/L). From these data, chylous reflux syndrome involving the pericardium and lung was diagnosed. An ultrasound examination showed obstruction of the left carotid segment of the thoracic duct:the internal diameter of the left cervical thoracic duct is about 2.3 mm, its terminal lumen is thin and the inner diameter is less than 1 mm. Lymphatic imaging by CT of the lower extremity (^99m^Tc-DX) showed that the lymphatic system of both lowerlimbs was unobstructed, and radioactive filling around the pericardium supported chylous effusion and continuous development of the upper thoracic duct.Magnetic resonance in mediastinum revealed that the thoracic duct from the tracheal carina to the diaphragm could be seen and that the lumen was slender. An abnormal tubular structure was seen around the carina and the bronchi on both sides, with that on the right being more obvious. The cervical portion of the thoracic duct was tortuous and appeared slightly tapered at the end. Direct lymphangiography showed that the contrast agent flowed slowly. The thoracic duct was slightly broadened in the lower and middle segments but broader in the upper segment. There was reflux of contrast agent into the left neck, left subclavian, and left bronchomediastinal lymphatic trunks. Contrast agent was deposited in the pericardium (Fig. 4). A lymphatic obstruction at the thoracic duct was noted but its cause was unclear.

During surgery, the end of the thoracic duct, ampulla, and various lymphatic trunks were found to be parceled and compressed by thicker fibrous tissue and a vascular sheath of the internal jugular vein. Chylous reflux was observed in the lymphatic tubes of the neck, bronchomediastinal, and subclavian trunks. Lymph fluid was not flowing freely into the blood. Thicker fibrous tissue and the vascular sheath were separated, yet the end of the thoracic duct remained compressed by the neck trunk. The thoracic duct and ampulla were at an angle owing to displacement by the chest wall lymphatic tubes, so lymphatic drainage was limited. Therefore the involved chest wall lymphatics were ligated, compression of the thoracic duct was relieved, and lymph flow returned to normal. Chylous reflux in the neck and subclavian trunks was significantly alleviated. After surgery, the patient felt severe tightness in her chest, and the pericardial effusion increased significantly for a week. There was a moderate amount of pleural effusion on the left and a small amount on the right. Pleural fluid on the left was aspirated and triglycerides were 12.63 mmol/L. Symptoms improved after pericardial and hydrothorax aspiration, and both cough and expectoration were diminished. The dimensions of the chylopericardium were as follows: apex, 18 mm; left rear, 37 mm; left, 28 mm; right front, 12 mm. The patient is being followed even now.

## Discussion and conclusions

Chyloptysis is the expectoration of chyle [9]; its rarity caused the patient depicted here to be misdiagnosed with recurrent pneumonia for 29 months, since the chyloptysis was not recognized and the pericardial effusion not aspirated. Another limitation was the difficulty in diagnosing the etiology of the reflux. Thus, to our knowledge, this is the first case in the literature regarding primary chyloptysis caused by mechanical compression and pulling of the thoracic duct with recurrent episodes of chyloptysis associated with chylopericardium. The etiology of the fibrous tissue around thoracic duct might be congenital, as there was no history of trauma or surgery. The obstruction was located near the end of thoracic duct, leading to the patient’s intrathoracic lymphatic reflux disorder. The obstruction induced chylopericardium and chyloptysis, but no symptoms of a systemic lymphatic reflux disorder were noted.

The diagnosis of chyloptysis is not difficult if the characteristic sputum is recognized. If chylous pleural or pericardial effusions accompany chyloptysis, diagnosis is easier. A cough producing white sputum occasionally slightly streaked with bloodcould indicate recurrent episodes but without bronchial infection, and an acellular sputum may indicate chyloptysis [9]. The production of chylous sputum is not always postprandial and not always associated with the intake of fatty foods. In addition to the quantification of cholesterol and triglycerides, lipoprotein electrophoresis is useful for confirming the chylous nature of the sputum. Chylous mucus in the airway can solidify overnight resulting in formation of chylous bronchial casts [5]. Plastic bronchitis could be formed by many compositions. Recognition of the characteristic appearance and differential diagnosis of mucus plugs will hopefully facilitate diagnosis and management [10, 11].

Chyloptysis is caused by chylous reflux from the thoracic duct into the lung; it can be caused by high peribronchial lymphatic pressure if the thoracic duct is obstructed above the midmediastinal level or may be due to incompetence of the lymphatic valves [1]. Obstruction of the thoracic duct can cause retrograde flow from the thoracic duct into the bronchomediastinal trunks and the peribronchial lymphatic plexus. When the peribronchial lymphatics are subjected to a circulatory overload of chyle plus lymph fluid from the lungs, the overloaded peribronchial and pulmonary lymphatic tubes become dilated and engorged. Mediastinal tumors, particularly lymphomas, are the main cause of mechanical obstructions of the thoracic duct. The main causes of chyloptysis is listed in Table 1. Chyloptysis is typically seen with lymphangiomatosis [12, 13], lymphangioleiomyomatosis(LAM) [3], thoracic lymphangiectasis [14], yellow-nail syndrome [9], Behçet’s disease [15], and iatrogenic injury [6, 16] (Table 1). Incompetence of the lymph valves is often congenital (lymphatic dysplasia) and can lead to chylous pleural effusion, chylous ascites, chyluria, and lymphedema of the lower extremities. In the present case, chyloptysis was due to rupture of the primary dilated lymphatic vessels, and subsequent reflux of thoracic lymphatics into the tracheobronchial tree caused by the compression of neighboring tissue. We found chylothorax after surgery but not before. We think that there was no bronchopleural fistula in the context of concurrent chylous pleural effusion before surgery. Compared with directly mechanical obstruction, chyloptysis in LAM can occur due to the generation of abnormal communication between lymphatic channels and the bronchial tree since LAM cells proliferate along the bronchial trees and gradually destroy the airway.

**Table 1:** The main causes of chyloptysis

obstruction of the thoracic duct	lymphatics-derived	lymphangiomatosis, lymphangioma,
		lymphangioleiomyomatosis
	compressed	mediastinal tumors
	iatrogenic injury	surgery, radiation, trauma
an abnormality of the lymphatics		lymphangiectasis,
incompetence of lymphatic valves		Bechet's disease
		lymphatic dysplasia
		(including yellow nail syndrome)
high venous pressure		coronary artery disease, untreated heart failure

Chyloptysis is primarily diagnosed by lymphangiography [17], contrast lymphangiography, or MRI. Lymphangiography is recommended to identify the anatomy and site of the lymphatic leak. Lymphoscintigraphy, which is noninvasive, has historically been the imaging modality of choice [18]. Contrast lymphangiography can be used to confirm anatomy, lymphangiectasia, and the site of the lymphatic leak; it is also helpful in planning surgical treatment. Three-dimensional MRI may offer extensive information about the anatomy of the lymphatic vasculature and the effects of lymphatic dysfunction on local structures and tissue composition [18]. HRCT is the most sensitive modality to identify the characteristic pathognomonic features of LAM, which are pulmonary cysts, ranging between a few mm and 1 cm in diameter with a thin wall with clear borders from the underlying normal parenchymal image, scattered evenly over all normal lung fields [19]. Fat content should be tested on the bronchial casts or bronchial alveolar lavage fluid. Additionally, a bronchoscopy should be performed to look for an irregular connection between the pulmonary and lymphatic systems.

Treatment for chyloptysis is focused on treating the primary cause and decreasing chyle formation. In most patients, symptoms can be relieved by eliminating the underlying cause or bypassing the thoracic duct [7]. A low-fat diet may also decrease the production of chyle [1]. Management of the bronchial casts includes bronchoscopic extraction, which may minimize the occurrence of inflammation [5, 20].

Chyloptysis is a rare but typical symptom of chylous disorders and is easy to misdiagnose. In this case mechanical compression and pulling of the thoracic duct might have been induced by a congenital disorder, causing recurrent chyloptysis accompanied by chylopericardium. Surgery was effective for the chyloptysis, although the chylopericardium could not be resolved, likely because this area does not absorb fluid well or another pathological process is ongoing.
